# Satellite image fusion to detect changing surface permeability and emerging urban heat islands in a fast-growing city

**DOI:** 10.1371/journal.pone.0208949

**Published:** 2019-01-02

**Authors:** Rajchandar Padmanaban, Avit K. Bhowmik, Pedro Cabral

**Affiliations:** 1 NOVA Information Management School (NOVA IMS), Universidade Nova de Lisboa, Campus de Campolide, Lisbon, Portugal; 2 Future Earth, Royal Swedish Academy of Sciences, Stockholm, Sweden; 3 Stockholm Resilience Centre, Stockholm University, Sweden; Universidade Federal de Uberlandia, BRAZIL

## Abstract

Rapid and extensive urbanization has adversely impacted humans and ecological entities in the recent decades through a decrease in surface permeability and the emergence of Urban Heat Islands (UHI). While detailed and continuous assessments of surface permeability and UHI are crucial for urban planning and management of landuse zones, they mostly involve time consuming and expensive field studies and single sensor derived large scale aerial and satellite imageries. We demonstrated the advantage of fusing imageries from multiple sensors for landuse and landcover (LULC) change assessments as well as for assessing surface permeability and temperature and UHI emergence in a fast growing city, i.e. Tirunelveli, Tamilnadu, India. IRS-LISSIII and Landsat-7 ETM+ imageries were fused for 2007 and 2017, and classified using a Rotation Forest (RF) algorithm. Surface permeability and temperature were then quantified using Soil-Adjusted Vegetation Index (SAVI) and Land Surface Temperature (LST) index, respectively. Finally, we assessed the relationship between SAVI and LST for entire Tirunelveli as well as for each LULC zone, and also detected UHI emergence hot spots using a SAVI-LST combined metric. Our fused images exhibited higher classification accuracies, i.e. overall kappa coefficient values, than non-fused images. We observed an overall increase in the coverage of urban (dry, real estate plots and built-up) areas, while a decrease for vegetated (cropland and forest) areas in Tirunelveli between 2007 and 2017. The SAVI values indicated an extensive decrease in surface permeability for Tirunelveli overall and also for almost all LULC zones. The LST values showed an overall increase of surface temperature in Tirunelveli with the highest increase for urban built-up areas between 2007 and 2017. LST also exhibited a strong negative association with SAVI. Southeastern built-up areas in Tirunelveli were depicted as a potential UHI hotspot, with a caution for the Western riparian zone for UHI emergence in 2017. Our results provide important metrics for surface permeability, temperature and UHI monitoring, and inform urban and zonal planning authorities about the advantages of satellite image fusion.

## 1. Introduction

Rapid urbanization has been globally a dominant driver of ecosystems and environmental degradation in the recent decades [1]. United Nations projected that two-thirds of the global population will live in urban areas by the year 2050 [2]. This will entail major landuse and landcover changes in urban areas, which will directly impact urban ecosystem services through a loss of agricultural and forested lands, and an increase of barren and impermeable built-up surface areas [3]. Loss of vegetation and increasing built-up surface areas may eventually affect climatic variability and thus lead to an increase in surface and air temperatures in urban areas [4–6].

Urban forests and vegetation control surface and air temperatures through shading and evapotranspiration [7]. According to the United States Environmental Protection Agency, shaded surfaces are, on average, 11–25°C cooler than unshaded surfaces, while evapotranspiration reduces peak summer temperatures by 1–5°C [8]. In contrast, impermeable built-up surface areas have a higher solar radiation absorption, and a greater thermal capacity and conductivity than the non-built-up areas [7,9]. Consequently, urban areas exhibit higher surface and air temperatures than surrounding rural areas [10]. Rapid urbanization and consequent expansion of impermeable built-up surface areas may thus lead to the emergence of urban heat islands (UHI), which have severe consequences for urban ecosystems and humans [4,11–13].

Monitoring the rate and extent of urbanization and consequent decrease in surface permeability and emergence of UHI provide essential information for averting their adverse impacts on urban residents and ecosystems [14]. Particularly, in fast growing cities like Tirunelveli, India, which are experiencing rapid and abrupt expansion due to extensive rural-urban migration and urban sprawl, UHI can emerge spontaneously through the loss of vegetation and expansion of impermeable surfaces [15]. Hence, they require detailed and continuous monitoring of landuse and landcover (LULC) changes. Emergence of UHI can be controlled and prevented through proper urban planning, management and regulations of landuse zones that are informed by detailed and continuous LULC change monitoring [16]. Monitoring anthropogenic LULC changes may also provide quantification of environmental processes and respective sustainable living standards in urban areas [17].

Remote sensing provides important tools for detailed and continuous monitoring of LULC changes in fast growing cities as well as for assessing expansion of impermeable surfaces and detecting emergences of UHI [10,11]. Remote sensing tools demonstrate clear advantage for monitoring and estimating spatiotemporal changes of LULC over conventional methods that are based on time consuming and expensive field studies combined with large scale aerial photography [18]. Hence, remote sensing techniques have been widely applied for assessing LULC changes, surface permeability and temperature, and detecting emergence of UHIs, in several regions of the world, e.g. Egypt [11], Eritrea [13], Germany [14] and Vietnam [19]. Particularly, the advent of high quality satellite imageries from multiple sensors for a certain location enables the fusion of those imageries to arrive at a combined image for that location [20]. Such combined images are substantially more detailed than images from individual sensors as they fuse images with diverse spatial and spectral resolutions and thus enable the detection of a diverse range of objects, which are often undetected through single sensor derived images [21–25]. However, monitoring of surface permeability and UHI emergence mostly involve time consuming and expensive field studies and single sensor derived aerial and satellite imageries.

Fused remotely sensed imageries provide important metrics for the quantification of LULC changes, surface impermeability and consequent increase in surface temperature in urban areas as well as for the assessment of the relationship between changes in surface permeability and surface temperature [10–12]. For instance, the Soil Adjusted Vegetation Index (SAVI) quantifies changes in vegetation cover and health in relation with soil moisture, saturation and color [26]. Hence, it has been widely used as an important proxy for surface permeability and also as an early warning metric for food security and ecological health [27]. Moreover, Land Surface Temperature (LST) is a metric for measuring the temperature of the interface between the earth’s surface and the atmosphere [16,28], which is often shaped by LULC and, particularly, vegetation cover [29]. Fused remotely sensed imageries provide considerably quicker continuous measurement of LST when compared with the conventional extrapolation of non-contiguous meteorological station measurements [30]. LST is also an important metric for the identification of the emergence and propagation of UHI [31]. Calculated SAVI can indicate climate change impacts in urban areas and hence, is associated with the changes in LST [26]. In general, areas with higher SAVI typically exhibit lower LST and vice-versa, given constant soil moisture and evapotranspiration capacity of the surface [32]. Overall, understanding the patterns of LULC, SAVI and LST changes, and their associations using fused remotely sensed imageries may provide quick and precise information crucial for urban ecosystem zoning and UHI control in fast growing cities like Tirunelveli [33,34].

This study quantified the LULC, SAVI and LST changes, as well as the relationship between SAVI and LST changes, in a fast-growing city, i.e. Tirunelveli, Tamilnadu, India, during a 11 years period, i.e. between 2007 and 2017. We fused satellite imageries from two different sensors, i.e. IRS-LISSIII and Landsat-7 ETM+, to arrive at combined high spatial resolution (23.5m of IRS P6-LISSIII) and high thermal band (30m of Landsat-7 ETM+) imageries for Tirunelveli. The objectives of our study are twofold: (i) To demonstrate the advantage of using fused imageries over non-fused single images through a comparison of image classification accuracies and (ii) to identify the potential zones for UHI emergence using a SAVI-LST combined metric for Tirunelveli in 2017.

## 2. Study area

**Fig 1:**
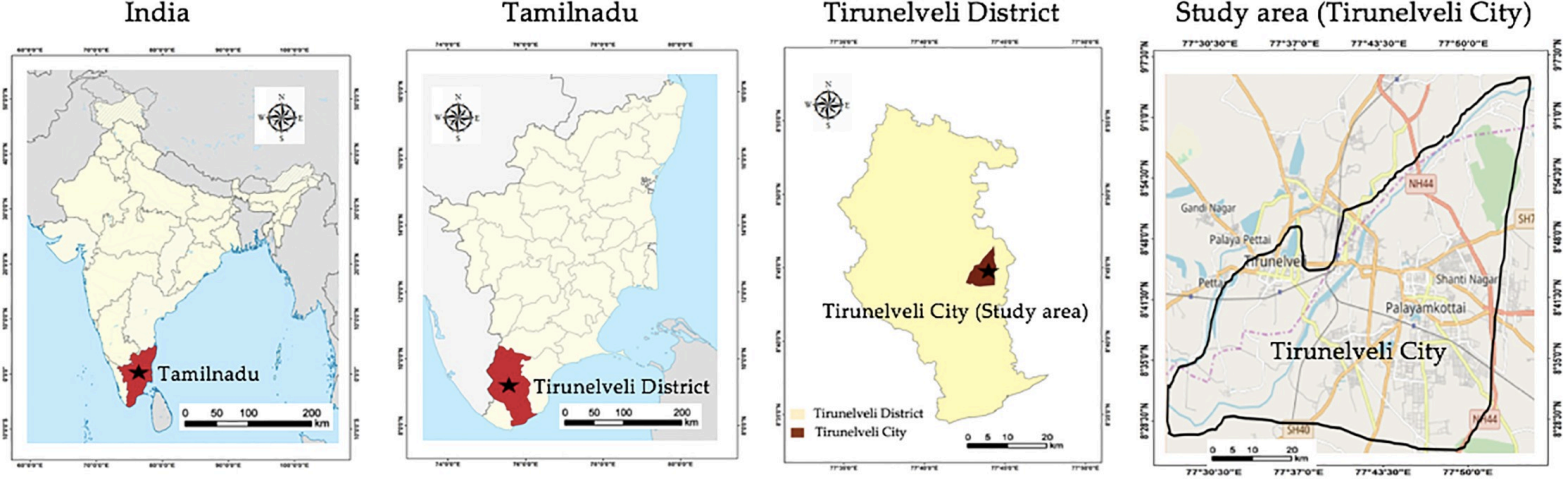
Geographic location and area of Tirunelveli city. The maps were generated using Google Maps.

Tirunelveli is one of the largest and oldest municipal corporations at Tirunelveli district in Tamilnadu state of India with a total population of 473,637 according to the 2011 census [35]. The city lies between 8°44’ and 9°30’of the Northern latitude, and 77°05’ and 78°25’ of Eastern longitude with an altitude of 47m above the mean sea level (Fig 1). Tirunelveli is situated on the East bank of Thamirabarani River, the major water course for domestic usage, power generation and irrigation in Tirunelveli and other neighboring cities (Tuticorin, Sankarankovil and Valliyur) [36].

The climate of Tirunelveli is dominantly tropical and receives rainfall in all seasons throughout a year [33]. The average annual rainfall during 2010–2016 was 947.6mm [37], with a contribution of 555.08mm and 189.6 mm rain from the North-East and South-West Monsoons, respectively. The average annual surface temperature of the city varies between 24.4°C and 34.6°C, with the lowest winter (November to February) and highest summer (March to June) temperatures of 27.1°C and 30.4°C, with an average precipitation of 127.7mm and 74.5mm, respectively [37].

Tirunelveli experienced a rapid and extensive urbanization and urban sprawl during the last two decades [15]. The city population has doubled during this time, which depicts Tirunelveli as one of the fastest growing cities in the India [34]. As a principal business hub of Southern India, Tirunelveli experienced a substantial immigration of people from neighboring cities and rural areas in search for better standard of lives, income and employments [15]. This has caused an uncontrolled expansion of the city and associated adverse effects on the city land, water and air [38]. According to the Centre for Agriculture and Rural development studies (CARDS), the rapid urbanization driven conversion of agriculture lands in Tirunelveli and surrounding districts have adversely impacted the region’s food security [39,40]. The rapid urbanization also entailed rapid industrialization leading to the establishment of more than 25 large-scale industries such as cement factories, cotton yarn manufacturers, calcium carbide production plants, sugar factories, cotton seeds oil refinery plants, brick factories, paper and flour mills, and several hundreds of small-scale industries. This, in turn, led to air pollution, water scarcity, degradation of vegetation, ecosystems fragmentation, floods and droughts [41].

We selected an area of 104.2 km^2^ covering the central area and periphery of Tirunelveli city (Fig 1). According to CARDS, this area has undergone the highest LULC conversion in Tirunelveli district between 2007 and 2017 [40]. Hence, we chose the years 2007 and 2017 for assessing LULC zones, SAVI and LST in Tirunelveli, as well as for quantifying their changes and detecting UHI emergence in our study.

## 3. Materials and methods

### 3.1 Data

We used Landsat Enhanced Thematic Mapper (ETM+) and Indian Remote Sensing Satellite Resourcesat-1—Linear Imaging Self-Scanning Sensor -3 (IRS LISS-III) images with 30 m and 23.5 m spatial resolutions, respectively, from June 2007 and June 2017. Freely available Landsat satellite data were downloaded from Unites States Geological Survey (USGS) gateway in GeoTiff format [42]. IRS-LISSIII data was purchased from the National Remote Sensing Centre (NRSC), Indian Space Research Organisation (ISRO) in GeoTiff format [43]. Daytime images from 11^th^ June (summer) were chosen for both years to obtain the least cloud coverage possible as well as to control for the seasonal homogeneity in plant phenology for LULC classification, and SAVI and LST calculation, and thus to exclude impacts of seasonal variation of plant phenology [44]. Landsat ETM+ and IRS-LISSIII were georeferenced using the World Geodetic System (WGS) 1984 and then projected to the Universal Transverse Mercator (UTM) coordinates [45,46]. The data has been geo-corrected and cropped to the study area (Fig 1).

### 3.2 Image pre-processing

**Fig 2:**
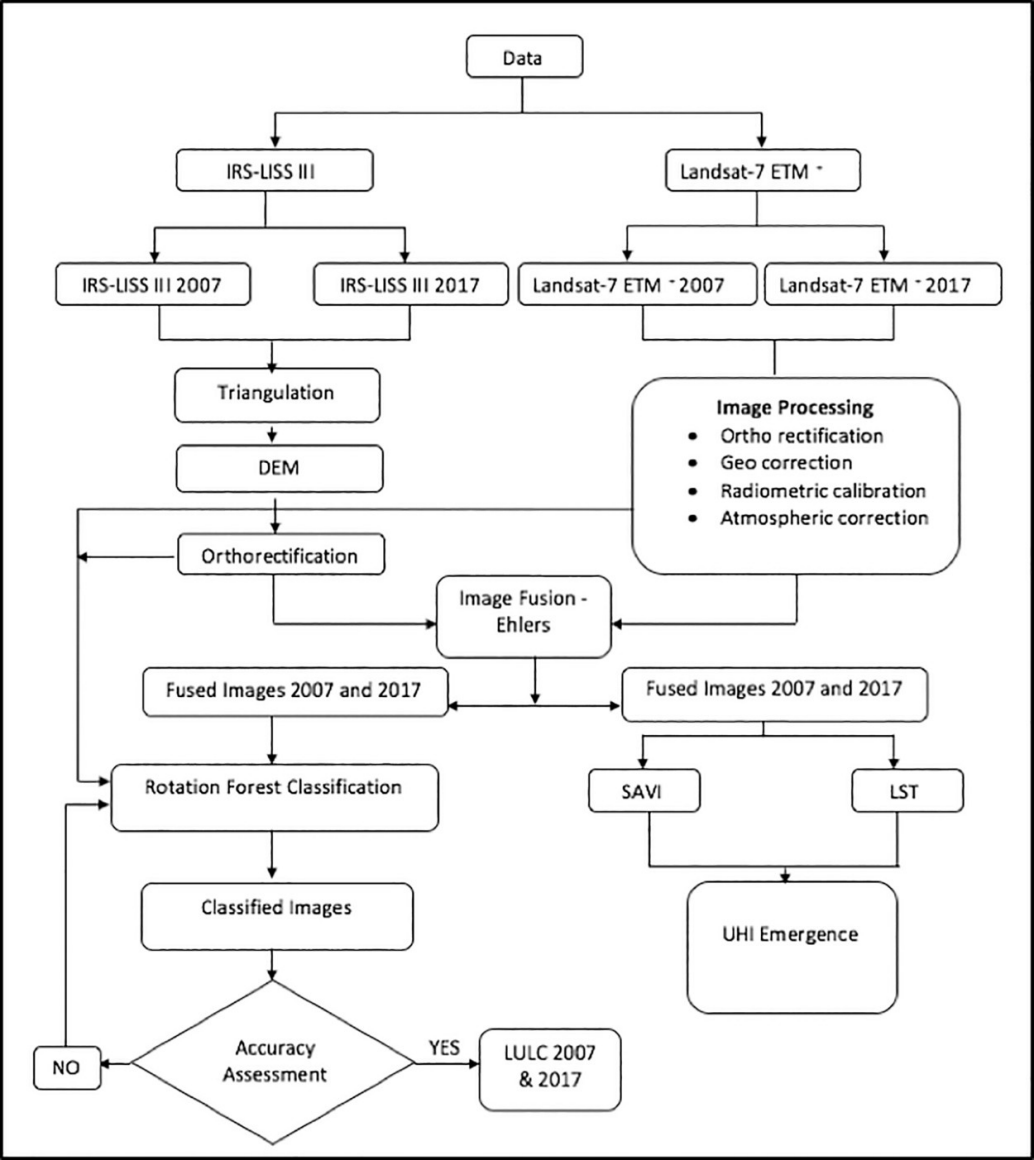
Methodological flow for landuse and landcover classification, surface permeability, surface temperature and urban heat islands emergence assessment. DEM: Digital Elevation Model; LULC: Landuse and Landcover; ETM: Enhanced Thematic Mapper; SAVI: Soil-Adjusted Vegetation Index; LST: Land Surface Temperature; UHI: Urban Heat Island.

We first pre-processed the Landsat ETM+ and IRS-LISSIII images, separately, for 2007 and 2017 (Fig 2). Triangulation and Digital Elevation Model (DEM) were generated for IRS-LISSIII images from each year to examine the land dynamics and prime variations [47]. Triangulation process for IRS-LISSIII was performed by fitting a second order polynomial in Leica Photogrammetry Suite (LPS) [48]. Then DEMs were generated using built-in image matching techniques [49]. DEMs were further edited using the built-in pit removal technique in LPS, where the abrupt elevational changes were identified [50]. The final DEMs of 2007 and 2017 were further orthorectified for LULC classification and analysis (Fig 2).

The Landsat ETM+ images were first geometrically corrected and orthorectified using the “georef” and “geoshif” functions of the “Landsat” package in R [51]. Then the orthorectified images were checked for scan line errors that occurred in the Landsat 7 ETM+ sensor from 2003 onward and consequently, influenced our images from 2007 and 2017 [52]. The missing data occurred due to scan line error were filled with the Landsat 7 Scan Line Corrector (SLC)-off Gap function in ERDAS Imagine (version 8.7) [46,53]. The SLC-off images were further rectified by mosaicking as recommended by USGS, and the residual gaps were filled using the histogram correction technique [52].

We performed radiometric and atmospheric corrections of the orthorectified and SLC-off Landsat ETM+ images. First, we transformed Digital Number (DN) integer values (0–255) in Landsat ETM+ images to at-satellite radiance values using the ETM+ radiometric calibration of Top-of-Atmosphere (TOA) radiance [53]. Then, we applied atmospheric correction to minimize the mismatch between surface reflectance and at-sensor reflectance [54]. The cloud, aerosol, and cirrus were identified and classified, and removed using Dark Object and Modified Dark Object Subtraction method [54]. Finally, to ensure the homogeneity of reflectance values for the analysis of surface permeability, invariant features in images from 2007 and 2017 were identified using the Pseudo-invariant features (PIF) function and subsequently corrected using a major axis regression [53]. The radiometric and atmospheric corrections were conducted in R environment [51].

### 3.3 Image fusion

The pre-processed IRS-LISSIII and Landsat 7 images were combined using the “Ehlers” image fusion technique [55,56]. Ehlers fusion works based on an Intensity-Hue-Saturation (IHS) transformation coupled with adaptive filtering in the Fourier domain to prevent the fused image from color distortion, which frequently occurs in conventional statistical or color transformation fusion methods (see [57] for details on Ehlers image fusion techniques). To avoid loss of information and further minimize color distortion, we also used all bands from IRS-LISSIII and Landsat ETM+ for the fusion process. The image fusion was performed using the “panSharpen” function of the “RStoolbox” package in R [58]. We maintained 30m resolution in fused images for further classification and indices calculation.

### 3.4 Image classification

We classified the fused images using a Rotation Forest (RF) machine learning algorithm [59]. Previous studies have demonstrated the higher accuracy levels of RF than other available methods for fused and non-fused image classification, such as GentleAdaBoost and Random Forest [60]. RF is based on an ensemble construction and is associated with a Decision Tree (DT), where each classifier is individually constructed [59]. The DT classifier is constructed following a five-fold process: (1) a K subset is randomly split from the feature set. The split subset are intersecting and disjoint, while we chose the disjoint subsets for a high diversity of features; (2) a Principal Component Analysis (PCA) is applied to each of the subsets to identify the variability information in the data; (3) undefined LULC classes are categorized; (4) the regular buoyancy for each class is computed; and (5) the label for each class is allocated to the one with the maximum buoyancy value [59].

We delineated eight LULC classes from the fused images of 2007 and 2017 using RF with the built-in DT classifier (see Table 1 for LULC classes definition). The delineation process includes the following steps in R [59] for training the DT classifier and image classification:

1. Build the stack for the fused raster data;
2. Divide the feature data set d into K feature subsets, each subset holds M = n/K number of feature;
3. Let F_i,j_ be the jth, j = 1,..,K, subset of features for L_i,_ and X_i,j_ be the features in F_i,j_ from X;
4. Select new training set from X_i,j_ randomly using a bootstrap algorithm;
5. Transform X_i,j_ to get the coefficient m_i,j_, …, m_i,j_, the size of m_i,j_ is M * 1;
6. Implement the following sparse rotation matrix R_i_, which is systematized with the above coefficients.
  $$Ri=[\begin{array}{cccc} mi,1,...,mi,1 & 0 & \ldots & 0\\ 0 & mi,2,...,mi,2 & .. & 0\\ . & . & . & .\\ . & . & \ddots & .\\ . & . & . & .\\ 0 & 0 & . & mi,k,...,mi,k \end{array}]$$ (1)
7. Rearrange matrix R_i_ to $R_{i}^{a}$ with respect to the initial feature set;
8. Train the classifiers in a parallel style;
9. Compute the confidence of the given data *χ* for each landuse class by an average combination method:
  $$\mu K(x)=\frac{1}{L}\sum_{i=1}^{L}\gamma i,k(\chi \;R_{i}^{a}),K=1,\ldots .,c$$ (2)
  Where $\gamma i,k(R_{i}^{a})$ is the probability produced by L_i_
10. Allocate *χ* to the landuse class with the highest confidence.
11. Transform the raster LULC classes into homogenized vector polygons. We selected the classification and regression tree (CART) transformation method, which is based on a decision tree algorithm and Gini index.
  $$Gini(t)=\sum_{i=1}^{c}p_{wi}\;(1-pwi)$$ (3)
  Where c is the number of LULC classes and *p*_*wi*_ is the probability of class *w*_*i*_ at node t.
  $$p_{wi}=\frac{n_{wi}}{N}$$ (4)
  Where N is the total number of training set samples and *n*_*wi*_ is the number of samples of class *w*_*i*_
12. Extract the DN values of polygon classes derived from CART;
13. Generate numbers of polygons cohering to the DN values;
14. Allocate color bands to the LULC classes.

**Table 1:** Landuse and landcover (LULC) classes definition.

No	LULC Classes	Definition
1	Barren land	Dry lands and non-irrigated
2	Crop land pasture	Agriculture lands, grazing area, coconut and banana farm
3	Fallow land	Non-plowed, dry farming area and real estate plots
4	Forest	Deciduous forest
5	Scrubs	Bushes and shrubbery
6	Urban	Roads, temples, and built-up areas
7	Water bodies	Rivers, lakes, open water, and ponds
8	Wetland	Marsh, bog, fen and swamp

We also applied the above image classification algorithm on the non-fused IRS-LISSIII and Landsat ETM+ images of 2007 and 2017 to compare the image classification accuracies between the fused and non-fused images.

### 3.5 Accuracy assessment

We assessed and compared the accuracy of classification between the fused and non-fused images of 2007 and 2017 [61]. Cartographic map of 2007 and classified Google Earth images of 2017 (as cartographic map wasn’t available) of the Tirunelveli city obtained from BHUVAN, ISRO India and Google Earth Engine (GEE), respectively, were used as reference images (ground truth) for the accuracy assessment of the classified LULC maps and comparison between the fused and non-fused images [62]. 75 Random pixels were generated from the classified LULC data and LULC values were extracted for those pixels for 2007 and 2017. Then, the LULC values were identified for the same pixels in the referenced images and compared with the LULC values of classified images. We employed the kappa coefficient as the accuracy indicator [61]. A kappa coefficient of more than 0.8 indicates a satisfactory accuracy of LULC maps, i.e., classified images are satisfactorily analogous to the reference data [63]. Kappa coefficients were computed for the classified fused and non-fused images in ERDAS Imagine (version 8.7) and compared. We also computed the producer and user accuracies of image classification through a confusion matrix [61].

### 3.6 Surface permeability assessment

We computed Soil-Adjusted Vegetation Index (SAVI) to assess the changes in surface permeability in Tirunelveli between 2007 and 2017. Generally, SAVI indicates vegetation coverage and health with respect to soil moisture, saturation and color, and thus accounts for the high variability of built-up and non-built-up land cover in urban areas [26,64]. SAVI also controls for the influence of soil brightness in Normalized Difference Vegetation Index (NDVI) and thus, minimizes soil brightness-related noise in vegetation coverage estimation [65]. Since coverage, brightness and health of vegetation are strongly associated with surface permeability, SAVI provides an important proxy for the identification of impermeable surfaces, particularly in urban areas [27]. We calculated SAVI using Eq (5) [66].

$$SAVI=\frac{\left(NIR-RED\right)}{\left(NIR+RED+L\right)}*(1+L)$$ (5)

Where, RED is the reflectance of the band 3 (RED band) and NIR is the reflectance value of the near infrared band (Band 4). L is the soil brightness correction factor. For dense vegetation and highly permeable surface areas, L = 0 and for vegetation scarce and impermeable surface areas, L = 1 [65]. Due to high dynamics of vegetation and built-up coverage in Tirunelveli (urban areas in general), L was set to 0.5 [66].

SAVI was computed for each pixel of the fused images from 2007 and 2017. We delineated five raster zones based on natural breaks in SAVI values of the pixels to distinguish among degrees of surface permeability, e.g. 0.54–1 and −1–0.08 zones indicated highly permeable surface with high density healthy vegetation and impermeable surface with low density unhealthy or no vegetation (mostly barren and fallow land, and built-up surfaces), respectively. We computed the area coverage of each soil permeability zone in 2007 and 2017 and calculated percentage changes in their coverage between 2007 and 2017. Areal average and standard deviation of SAVI were also computed for each LULC class in 2007 and 2017.

### 3.7 Land surface temperature measurement

We calculated Land Surface Temperature (LST) index for each pixel of the fused images from 2007 and 2017 to measure the radiative skin temperature of the surface and its features, which depends on the optical brightness and reflectance of the surface (Albedo) [32]. Generally, bare soil and built-up settlements with low SAVI exhibit high Albedo whereas dense vegetation with high SAVI exhibits low Albedo and hence, low radiative skin temperature [67]. Thus, LST indicates climatic variability across vegetation and urban settlements associated with the degree of surface permeability [68]. LST for each pixel was calculated using Eq (6) according to the Landsat user’s hand book, in which the digital number (DN) of thermal infrared band is converted into spectral radiance (Lλ) [69,70].

Lλ={LMAX−LMIN÷QCALMAX−QCALMIN}*DN−1+LMIN} (6)

Where,

*LMAX =* the spectral radiance that is scaled to *QCALMAX* in W/(m2 *sr *μm)

*LMIN =* the spectral radiance that is scaled to *QCALMIN* in W/(m2 *sr*μm)

*QCALMAX* **=** the maximum quantized calibrated pixel value (corresponding to *LMAX*) in DN = 255

*QCALMIN* = the minimum quantized calibrated pixel value (corresponding to *LMIN*) in DN = 1

Raster maps of the LST index were computed for 2007 and 2017 from the fused satellite images and compared to assess changes in surface radiant temperatures in Tirunelveli between 2007 and 2017. To be coherent with SAVI classes, we delineated five LST raster zones based on natural breaks and computed their area coverage in 2007 and 2017. The average and standard deviation of LST for each LULC class were also computed. We also quantified the association between surface permeability and temperature through a Spearman raster correlation analysis between SAVI and LST for entire Tirunelveli also for the classified LULC zones.

### 3.8 Emergence potential for Urban Heat Islands

We quantified the emergence potential of Urban Heat Islands (UHI) in Tirunelveli using a combined metric computed from LST and SAVI in 2017 [14]. Generally, impermeable surface areas with lower SAVI exhibit higher solar radiation absorption, and a greater thermal capacity and conductivity, and consequently exhibit higher potential for UHI emergence, and vice-versa [9,71]. Moreover, areas with high surface temperature (LST) exhibit higher number of daily high degree-hours and lower differences between daily maximum and minimum temperatures, and thus also exhibit higher potential for UHI emergence, and vice-versa [72]. Hence, we first coded the five SAVI and LST classes from 1 to 5 in descending and ascending orders, respectively. Subsequently, we sum aggregated the recoded SAVI and LST class values for each pixel to compute the combined metric for UHI emergence potential. Pixels with higher combined UHI metric value indicated higher potential for UHI emergence and vice-versa. Finally, we delineated the zones with high UHI emergence potential in Tirunelveli.

## 4. Results and discussion

### 4.1 Landuse and landcover changes

**Fig 3:**
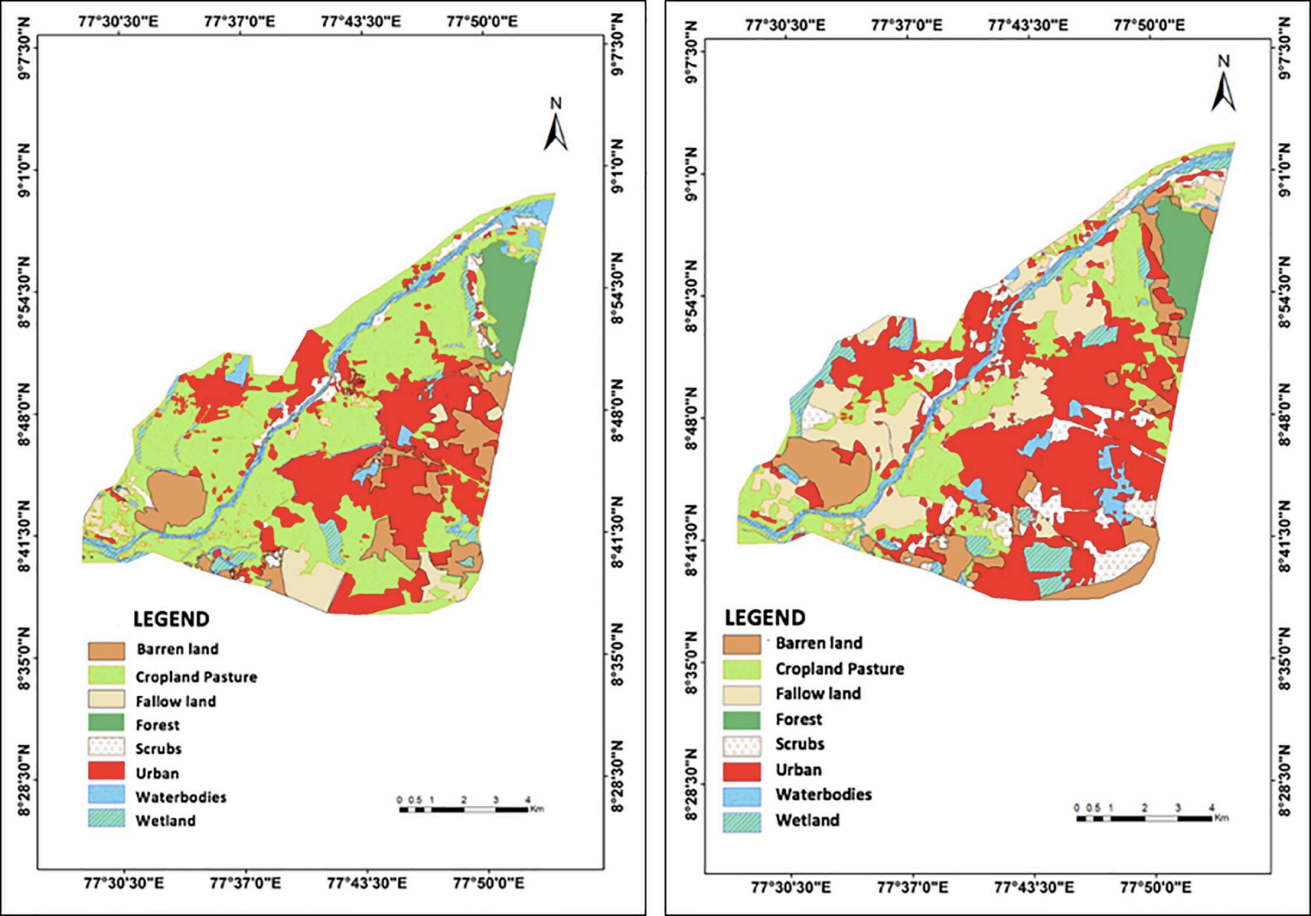
Classified landuse and landcover (LULC) maps of Tirunelveli city for 2007 (a) and 2017 (b).

The LULC maps of 2007 and 2017 show that the Tirunelveli city has undergone a rapid urbanization at an average rate of 4% between 2007 and 2017, with a 32% total increase in the coverage of urban built-up areas (Fig 3, Table 2). Fertile cropland pastures have been substantially converted (59% decline between 2007 and 2017) into fallow lands (mostly real estate plots, 178% increase between 2007 and 2017) and fallow lands (transitioning into built-up areas, 6% increase between 2007 and 2017). Forested areas in the Northeastern part of the city decreased by 12% whereas the bushes and shrubbery covered infertile areas increased by 164% throughout the city between 2007 and 2017. The Western riparian part of the city has undergone the most expensive LULC conversion from cropland pasture to fallow lands and built-up areas (Fig 3), which is in line with the findings of CARDS [40]. Although the wetland and waterbodies showed an aggregate increase by 35% between 2007 and 2017, forest cover and vegetation exhibited substantial decrease and conversion into urban areas (Fig 3, Table 2). These results are in line with [3], which estimated a rapid urbanization and urban sprawl in the Tirunelveli city between 2007 and 2017.

**Table 2:** Change in the area coverage of the landuse and landcover (LULC) classes between 2007 and 2017.

Classes	2007 (km^2^)	2017 (km^2^)	Change (%)
Barren land	10.07	10.69	6.15
Cropland pasture	45.42	18.43	-59.42
Fallow land	5.79	16.11	178.23
Forest	1.78	1.56	-12.35
Scrubs	3.37	8.89	163.79
Urban	27.85	36.73	31.88
Water bodies	3.84	4.25	10.67
Wetland	6.08	7.54	24.01
**Total**	**104.2**	**104.2**	

We obtained kappa coefficient values of 0.84 and 0.83 with an overall accuracy value of 86% and 85% for the LULC classification for 2007 and 2017, respectively, using fused images (Table 3). In contrast, the average kappa coefficient and overall accuracy values for LULC classification using non-fused images for 2007 and 2017 were considerably lower, i.e. 0.72 and 0.75, 71% and 74%, respectively. Hence, the accuracy of LULC classification using fused images was considerably higher than the LULC classification using non-fused images because of the substantially higher spatial resolution and number of bands available in fused images than non-fused images [25]. These results are also in line with [23].

**Table 3:** Accuracy assessment results for the landuse and landcover classification using fused images of 2007 and 2017.

Classes	2007		2017	
	Producer Accuracy	User Accuracy	Producer Accuracy	User Accuracy
**Barren land**	82.01	83.12	87.12	86.20
**Crop land and pasture**	84.15	87.14	82.56	83.21
**Fallow land**	82.34	89.16	87.32	92.35
**Forest**	84.15	90.12	84.51	87.18
**Scrubs**	88.11	84.21	83.15	82.13
**Urban**	91.32	89.34	88.29	96.07
**Waterbodies**	85.21	87.43	79.45	81.32
**Wetland**	89.01	91.03	84.56	89.04
**Overall accuracy**	**85.75**		**84.62**	
**Kappa**	**0.84**		**0.83**	

### 4.2 Changes in soil permeability and surface temperature

**Fig 4:**
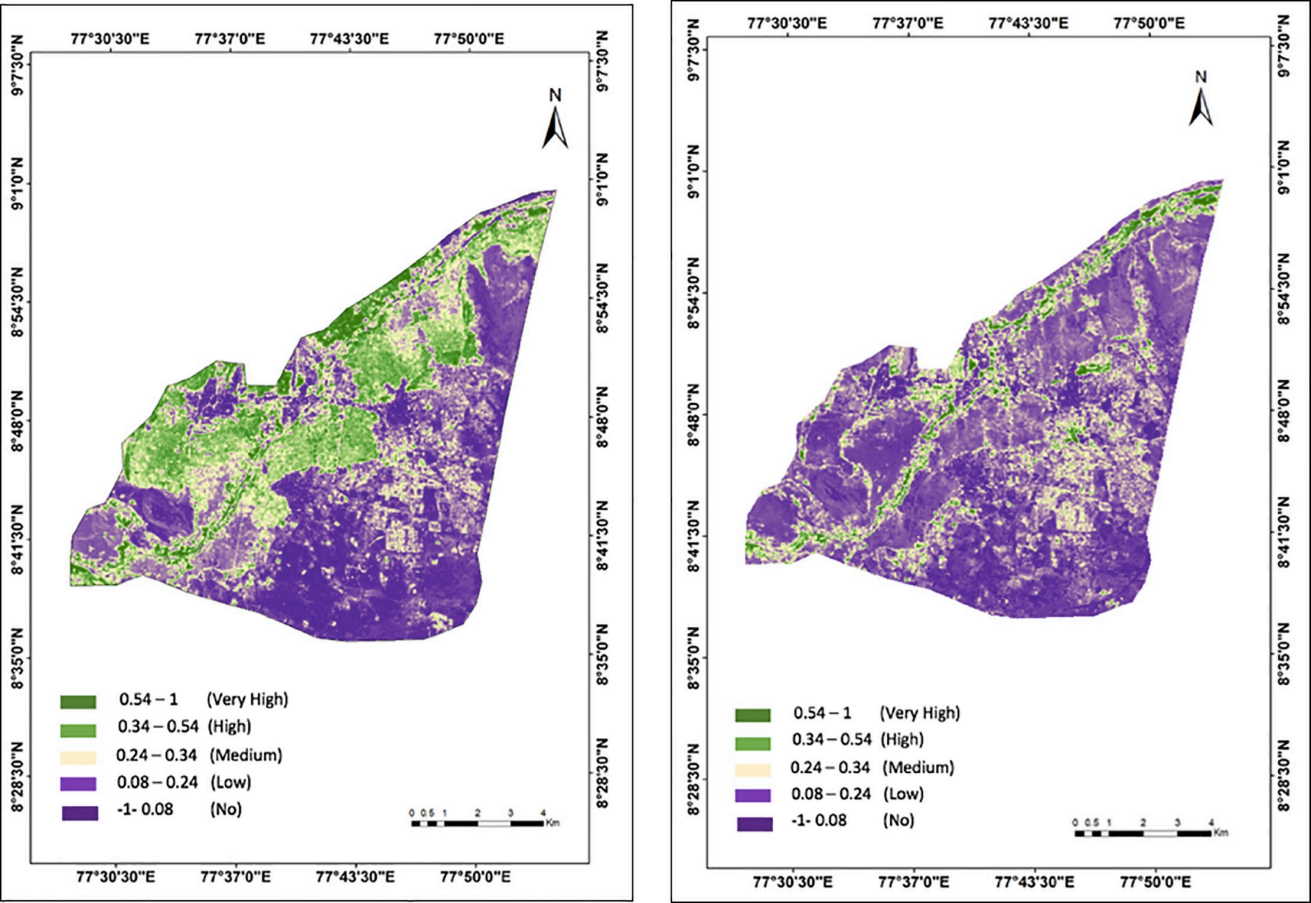
Soil-Adjusted Vegetation Index (SAVI) (surface permeability) maps of the Tirunelveli city in (a) 2007 and (b) 2017.

We observed a substantial decrease (58% on average) in the area coverage of permeable surfaces (SAVI values 0.08–1) while a substantial increase (33% on average) in the area coverage of impermeable surfaces (SAVI values -1-0.08) in Tirunelveli between 2007 and 2017 (Fig 4 and Table 4). The riparian zone at the Western part of Tirunelveli, which experienced the most extensive LULC conversion (Fig 3), also undergone the highest decline in highly and medium permeable surfaces (SAVI values 0.34–1) with dense vegetation between 2007 and 2017, i.e., the average SAVI value decreased from 0.54 to 0.08 (Fig 4). In general, the highly (SAVI 0.54–1) and medium (0.34–0.54) permeable zones undergone the highest decline, i.e. more than 87%, in Tirunelveli between 2007 and 2017 (Fig 4 and Table 4). Conversely, impermeable surface zones (SAVI values -1-0.08) exhibited a substantial increase, mostly around the built-up Southeastern part of Tirunelveli.

**Table 4:** Changes in the coverage of soil permeability (indicated by soil-adjusted vegetation index (SAVI)) classes in Tirunelveli between 2007 and 2017.

SAVI Classes	Surface permeability	Total Area Coverage (Km^2^)		Change in area coverage (%)
		2007	2017	
0.54–1	Very high	12.6	1.64	-86.98
0.34–0.54	High	8.34	0.87	-89.56
0.24–0.34	Medium	5.67	3.13	-44.79
0.08–0.24	Low	11.76	10.54	-10.37
-1-0.08	No	68.15	90.34	32.56

Generally, we observed high areal average SAVI values, i.e. high surface permeability, for the LULC zones with vegetation cover, e.g. cropland and scrubs, in contrast to the low SAVI values for built-up LULC areas, e.g. urban and barren land (Table 5). The average SAVI values for all LULC zones decreased between 2007 and 2017 apart from the Wetlands (Table 5). The highest average changes in SAVI values were observed for the barren and fallow lands (Table 5). Fallow land also represents the LULC class that has undergone the highest transition (178%) into real estate plots and built-up areas, i.e. urbanization (Table 2). This indicates that the extensive urbanization has adversely affected the soil permeability in Tirunelveli between 2007 and 2017, which is in line with [64]. Although the area coverage by water bodies has increased by 11% between 2007 and 2017 (Table 2), the permeability of the surface beneath also decreased for this LULC class (Table 5), indicating marginal or no improvement of soil permeability through anthropogenic development of water courses [65].

**Table 5:** Areal average SAVI values for the LULC zones.

LULC zones	2007	Standard deviation (±)	2017	Standard deviation (±)
Barren land	0.09	0.59	-1	0.70
Cropland pasture	0.42	0.13	0.29	0.08
Fallow land	0.08	0.48	-1	0.61
Forest	0.24	0.03	0.21	0.04
Scrubs	0.54	0.14	0.34	0.12
Urban	0.16	0.10	0.08	0.19
Waterbodies	0.97	0.24	0.54	0.37
Wetland	0.24	0.11	0.34	0.17

**Fig 5:**
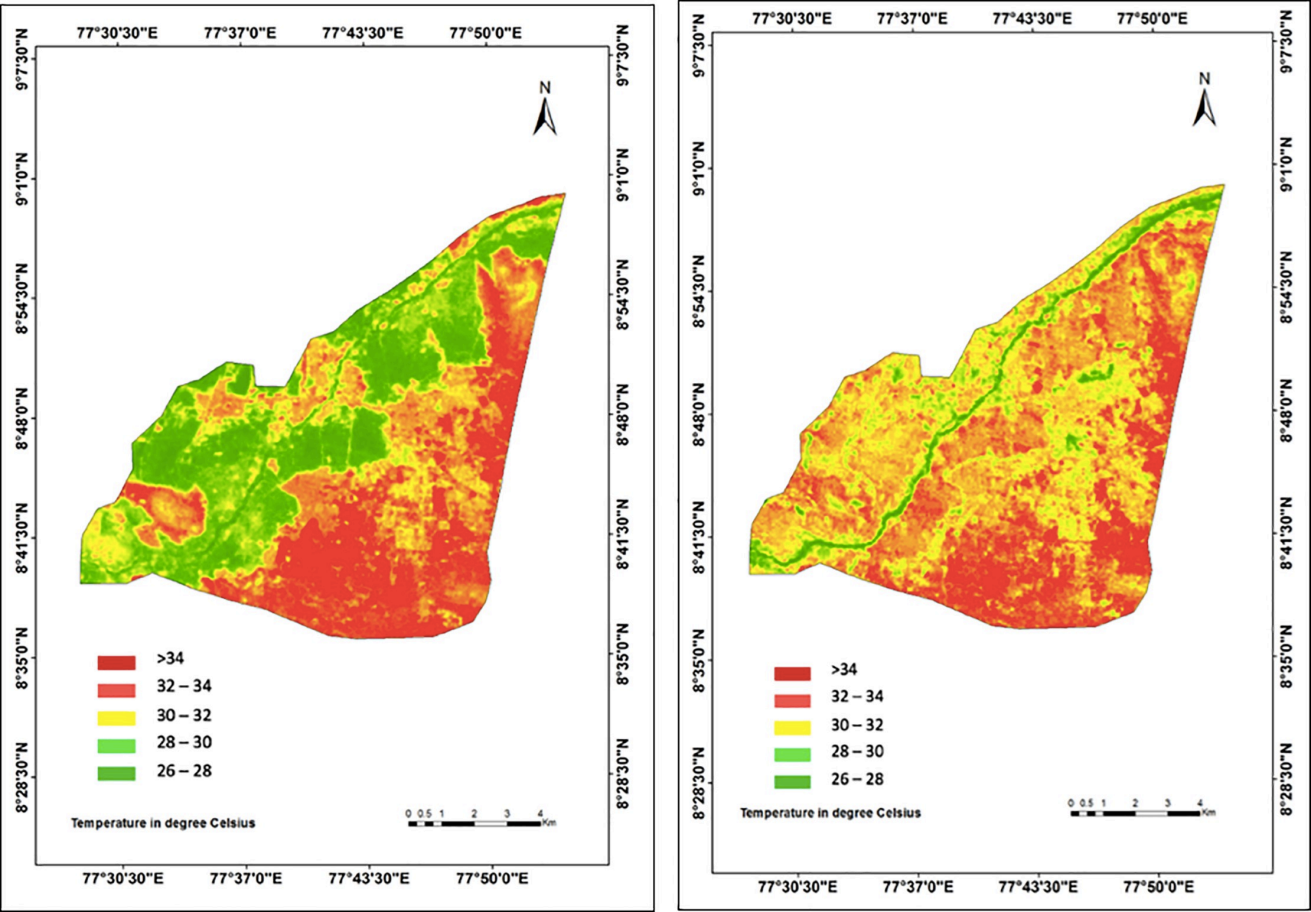
Land Surface Temperature (LST) maps of the Tirunelveli city for (a) 2007 and (b) 2017.

The overall climatic impact of extensive LULC conversion and decrease in surface permeability was evident by an average increase of LST by 1.3°C in Tirunelveli city between 2007 and 2017 (Fig 5 and Table 6). Particularly, the Western riparian zone, which has undergone the highest conversion of LULC and highest decrease in SAVI, also experienced the highest increase in LST, i.e. 4°C on average from 28°C to 32°C, between 2007 and 2017 (Fig 5 and Table 6). LST zone 30–32°C exhibited the highest increase in area coverage (165%) whereas the highest decrease was observed for the coverage of LST zone 26–28°C (64%) (Table 6). Overall, the low temperature zones (26–30°C) showed a decreasing coverage whereas the high temperature zones (more than 30°C) exhibited an increasing coverage in Tirunelveli between 2007 and 2017 due to extensive LULC conversion and surface permeability deterioration (Table 6), which is also in line with [69].

**Table 6:** Statistics of the area between 2007–2017 with corresponding changes in LST.

LST Classes (^0^C)	Area Coverage (km^2^)		Change in area coverage (%)
	2007	2017	
26–28	28.46	10.21	-64.12
28–30	19.97	7.21	-63.89
30–32	10.43	27.69	165.48
32–34	14.77	18.20	23.22
>34	30.57	40.89	33.75

In general, we detected higher LST values for the LULC zones with lower vegetation cover, e.g. barren and fallow land, and urban built-up areas, and with lower surface permeability (lower SAVI), and vice-versa, which is in line with [70] (Table 7). The highest areal average LST of above 34°C was observed for the urban built-up areas and barren lands in 2007, which has increased to above 36°C in 2017. In contrast, the lowest areal average LST of below 27°C was observed for croplands, fallow lands, water bodies and wetlands in 2007, which has also increased to below 28°C in 2017. The highest increase in areal average LST, i.e. 2.4°C, was observed for the urban area and barren lands (Table 7). Areal average LSTs of wetland and waterbodies also exhibited an increase of 1°C between 2007 and 2017 (Figs 4 and 5 and Table 7). Moreover, the deciduous forest area exhibited a decrease (0.03) and an increase (0.3°C) in the areal average SAVI and LST, respectively, between 2007 and 2017, indicating the adverse impact of overall rapid and extensive urbanization in Tirunelveli (Tables 5 and 7).

**Table 7:** Average land surface temperature (LST) in degrees Celsius by landuse and landcover cover (LULC) zones.

LULC zones	2007	Standard deviation (±)	2017	Standard deviation (±)
Barren land	34.106	5.32	36.604	7.92
Cropland pasture	26.861	5.89	27.112	6.32
Fallow land	34.127	3.21	35.821	6.87
Forest	32.153	3.15	32.462	3.91
Scrubs	26.242	6.13	27.357	7.44
Urban	34.196	1.11	36.620	2.73
Water bodies	26.824	6.98	27.868	8.03
Wetland	26.291	8.44	27.731	9.34

Note that we obtained lower SAVI (lower surface permeability) and higher LST (higher surface temperature) values for the forested area at the Northeastern part of Tirunelveli than other vegetated areas, i.e. cropland pastures and scrubs (Tables 5 and 7). This is because the deciduous forest of Tirunelveli sheds its leaves completely during summer (March—June) [34]. During this season, the surface of the dry forest receives the least precipitation with no other sources of irrigation, and absorbs the highest solar radiation with the highest temperature of the year [73]. Consequently, this dry decidous forest area exhibits relatively lower surface permeability and higher surface temperature than other vegetated areas during July in our study (Figs 4 and 5, Tables 5 and 7). Similar climatic responses of decidous forest were observed in summer by other studies investing impacts of climatic changes on forests [73]. Nevertheless, this deciduous forest area exhibited a decrease (0.03) and an increase (0.3°C) in the areal average SAVI and LST, respectively, between 2007 and 2017, indicating the adverse impact of overall rapid and extensive urbanization in Tirunelveli (Tables 5 and 7), which is in line with [15].

### 4.3 Urban heat Islands emergence

We observed a negative correlation between LST and SAVI overall, as well as by the LULC zones (Table 8). The overall correlation coefficients obtained for entire Tirunelveli in 2007 and 2017 were -0.24 and -0.72, respectively (both statistically significant at *p* ≤ 0.01). Urban built-up areas exhibited the highest correlation coefficients in 2007 and 2017, along with the highest increase in correlation coefficient values (Table 8). In general, LULC zones with lower surface permeability exhibited higher correlation coefficient values, and vice-versa. This indicates that a decrease in surface permeability entails an increase in surface temperature [68] and hence, exhibit a high potential for the emergence of UHIs [74].

**Table 8:** Spearman correlation between soil-adjusted vegetation index (SAVI) and land surface temperature (LST) by landuse and landcover (LULC) zones. All correlation coefficients are statistically significant at *p* ≤ 0.01.

LULC zones	Correlation coefficients		*p*-values
	2007	2017	
Barren land	-0.19	-0.25	0.0023
Cropland pasture	-0.11	-0.21	0.0049
Fallow land	-0.17	-0.27	0.0031
Forest	-0.07	-0.13	0.0012
Scrubs	-0.11	-0.13	0.0015
Urban	-0.29	-0.52	0.0062
Water bodies	-0.14	-0.16	0.0017
Wetland	-0.13	-0.25	0.0037

**Fig 6:**
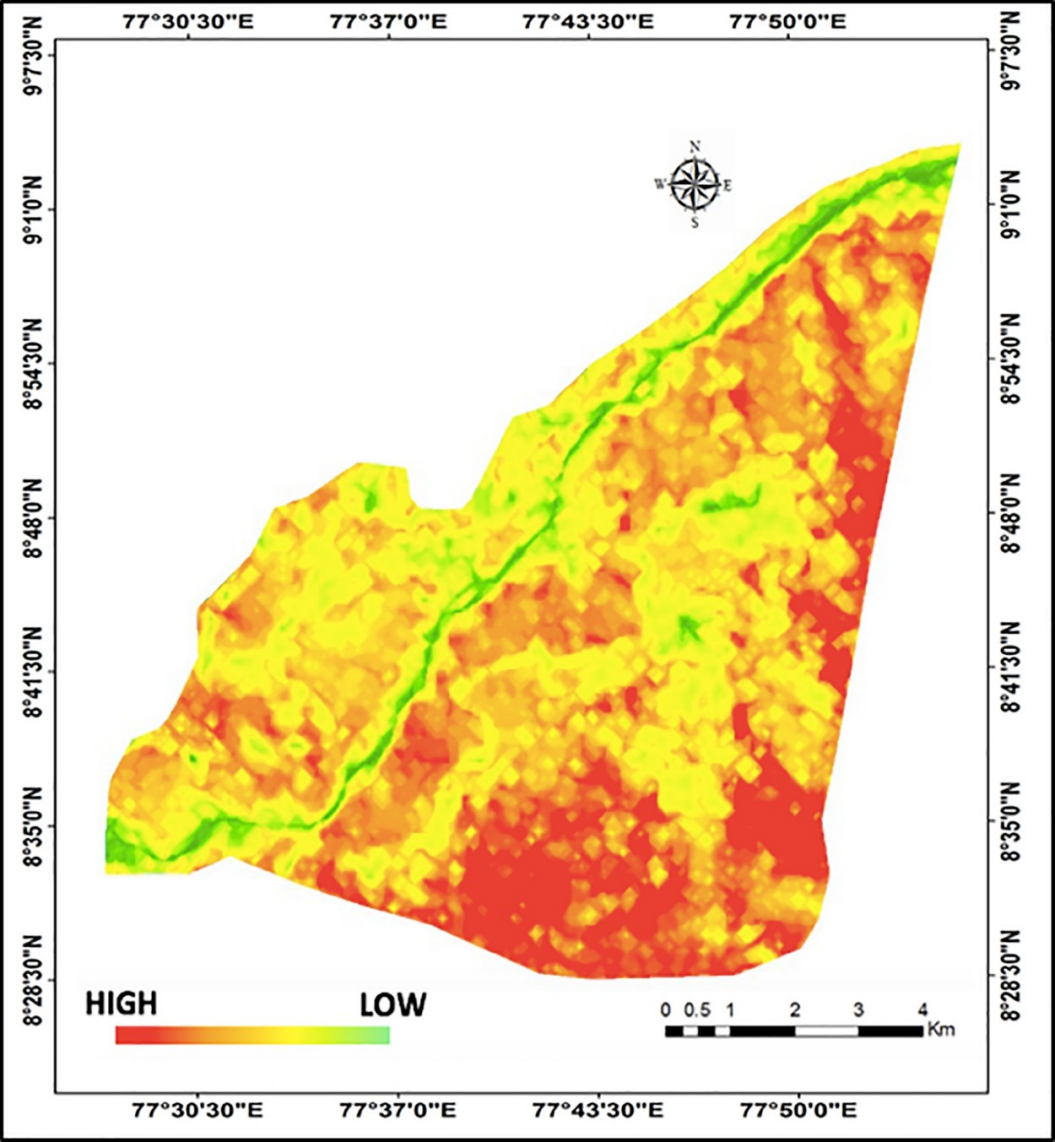
UHI emergence potential map of Tirunelveli city for 2017.

The emergence potential for UHI was high for the Eastern periphery of Tirunelveli in 2017 with the highest potential for the urban built-up areas at the Southeastern part (Fig 6). The Western riparian zone, which has undergone the highest LULC transition from cropland to barren and fallow lands, and urban built-up areas, also exhibited high emergence potential for UHI (Fig 6). Consequently, we suggest the Southeastern built-up areas in Tirunelveli as a potential UHI hotspot, while a caution for the Western riparian zone for UHI emergence that requires continuous and detailed monitoring. The waterbodies and wetlands, however, showed the lowest potential for UHI emergence, proving the importance of including waterbodies and greenspaces into urban planning to prevent the emergence of UHI [68].

Note that our analysis is limited to daytime imageries due to the unavailability of nighttime imageries and hence, did not measure nighttime temperature to determine the difference between minimum and maximum surface temperatures. This might affect the accuracy of UHI emergence detection using our SAVI-LST metric. However, since ours is a study on surface temperature and not air temperature, the variation between daytime and nighttime temperatures is marginal due to nighttime surface radiation [69]. Moreover, we used SAVI as an additional surrogate of LST, which provided important proxies for mean, range and variance of surface temperatures [64]. Furthermore, previous studies accurately detected and delineated UHIs based on only daytime imageries [14,31,75]. Hence, we suggest that our SAVI-LST metric is sufficiently robust for detecting UHIs emergence although nighttime imageries should be included when available.

## 5. Concluding remarks

We demonstrated the advantage of using fused satellite imageries combining multiple sensors in detecting and monitoring changes in land surface permeability and temperature and emergence of Urban Heat Island (UHI) in fast growing cities like Tirunelveli. Future studies should fuse higher temporal and spectral resolution imageries than the ones used in our study to provide a continuous, seasonal and more detailed assessment of Landuse and Landcover (LULC), Soil-Adjusted Vegetation Index (SAVI) and Land Surface Temperature (LST) changes, and UHI emergence in Tirunelveli [14].

The UHI emergence potential, which was computed by aggregating SAVI and LST provide important metrics for the identification and quantification of UHI zones. These metrics can be integrated in sophisticated UHI detection models for a more accurate and precise identification and quantification of UHI [75].

We suggest that urban landuse measures and zonal planning should be informed by detailed and continuous RS and GIS based assessment of LULC, SAVI and LST [26,67]. Possible measures include the conservation of agriculture and forested lands, and proper management of the reclamations of barren lands to pasture lands to avoid the decrease in surface permeability and ecosystem fragmentation [76]. Urban expansion should include provision of water bodies and afforestation to preserve surface moisture, permeability and radiative capacity, and thus to prevent the increase in surface temperature [71].
